# The LncRNA signature associated with cuproptosis as a novel biomarker of prognosis in immunotherapy and drug screening for clear cell renal cell carcinoma

**DOI:** 10.3389/fgene.2023.1039813

**Published:** 2023-01-23

**Authors:** Lishuo Zhang, Longjiang Di, Jinhui Liu, Xianli Lei, Maoli Gu, Wenjing Zhang, Yufu Wang

**Affiliations:** ^1^ Department of Urology, The First Affiliated Hospital of Harbin Medical University, Harbin, Heilongjiang, China; ^2^ College of Basic Medicine, Southern Medical University, Guangzhou, Guangdong, China; ^3^ The Second Affiliated Hospital of Harbin Medical University, Harbin, Heilongjiang, China; ^4^ Harbin Medical University, Harbin, Heilongjiang, China

**Keywords:** clear cell renal cell carcinoma, long non-coding RNA, cuproptosis, prognosis, immunotherapy

## Abstract

Cuproptosis is a new form of cell death, the second form of metal ion-induced cell death defined after ferroptosis. Recently, cuproptosis has been suggested to be associated with tumorigenesis. However, the relationship between cuproptosis and patient prognosis in clear cell renal cell carcinoma (ccRCC) in the context of immunotherapy remains unknown. The aim of this study was to investigate the correlation between cuproptosis-related long non-coding RNA (lncRNA) and ccRCC in terms of immunity as well as prognosis. Clinical information on lncRNAs associated with differences in cuproptosis genes in ccRCC and normal tissues was collected from The Cancer Genome Atlas (TCGA) dataset. Univariate Cox regression was used to screen lncRNAs. A total of 11 lncRNAs closely associated with cuproptosis were further screened and established using the least absolute shrinkage and selection operator (LASSO) algorithm and multivariate Cox regression, and the samples were randomly divided into training and test groups. A risk prognostic model was constructed using the training group, and the model was validated using the test group. We investigated the predictive ability of the prognostic risk model in terms of clinical prognosis, tumor mutation, immune escape, immunotherapy, tumor microenvironment, immune infiltration levels, and tumor drug treatment of ccRCC. Using the median risk score, patients were divided into low and high-risk groups. Kaplan-Meier curves showed that the overall survival (OS) of patients in the high-risk group was significantly worse than low-risk group (*p* < 0.001). Receiver operating characteristic (ROC) curves further validated the reliability of our model. The model consistently and accurately predicted prognosis at 1, 3, and 5 years, with an AUC above 0.7. Tumor cell genes generally precede morphological abnormalities; therefore, the model we constructed can effectively compensate for the traditional method of evaluating the prognosis of patients with renal cancer, and our model was also clinically meaningful in predicting ccRCC staging. In addition, lower model risk scores determined by mutational load indicated a good chance of survival. The high-risk group had greater recruitment of immune cells, while the anti-immune checkpoint immunotherapy was less efficacious overall than that of the low-risk group. Tumor and immune-related pathways were enriched, and anti-tumor agents were selected to improve the survival of ccRCC. This prognostic risk model is based on the levels of cuproptosis-associated lncRNAs and provides a new perspective in the clinical assessment and precise treatment of ccRCC.

## 1 Introduction

Renal cell carcinoma (RCC) is a relatively rare malignancy of the urinary tract, accounting for approximately 3% of all malignancies. In the urinary system, it is second only to prostate and bladder cancer in terms of incidence (Sung et al., 2021), with a late 5-year survival rate of only 12%. RCC is the deadliest urological cancer (Padala et al., 2020). Based on histological and molecular features, RCC includes a variety of subgroups. Among them, the most notable is renal clear cell carcinoma (ccRCC), which accounts for about 70%–80% of renal cell carcinomas (Srigley et al., 2013). The absence of a specific presentation makes the diagnosis of ccRCC difficult and its treatment ineffective. Even when surgical resection is used for limited-spread ccRCC, recurrence or metastasis occurs in about 30% of patients. Metastatic ccRCC is even more resistant to conventional therapies, resulting in poor patient outcomes (Rabjerg, 2017). The clinical prognosis of ccRCC is generally poor; tumor genetics and immunology need to be explored in order to present possible novel therapies. Avenues with possible therapeutic and prognostic utility include mRNA-lncRNA-miRNA networks, cell death patterns, and other mechanisms.

Copper is a biological element indispensable for the human body. Abnormal copper homeostasis can affect tumor progression through various mechanisms, such as apoptosis, autophagy, reactive oxygen species accumulation, and proteasomes (Ge et al., 2022; Jiang et al., 2022). Recent studies have suggested that copper dominates a specific form of cell death known as cuproptosis, the youngest member of the cell death field, and the second metal ion-induced form of cell death defined after ferroptosis (Kahlson and ScottDixon, 2022). Direct binding of copper to the lipid acylated components of the tricarboxylic acid (TCA) cycle results in lipid acylated protein aggregation and subsequent loss of iron-sulfur cluster proteins, leading to proteotoxic stress and, ultimately, cell death. A recent study screening 489 human cancer cell lines demonstrated that cuproptosis may be associated with tumor growth, multiplication, and invasion; thus, exploring the great potential of cuproptosis as a new avenue for future tumor-targeted therapy is needed (Zheng et al., 2014). However, the impact of cuproptosis on ccRCC prognosis is unclear, and a comprehensive understanding of ccRCC cellular cuproptosis, including the relationship between cuproptosis lncRNA and the tumor immune microenvironment, is still lacking.

Long non-coding RNA (lncRNA) is a heterogeneous set of non-protein-coding transcripts that are greater than 200 nucleotides in length (Kopp and Mendell, 2018; Bridges et al., 2021). During tumor progression, lncRNA can act as an oncogene or as a suppressor, controlling tumor proliferation, differentiation, invasion, and metastasis (Huarte, 2015). Its effects are mainly involved in regulating the transcription and translation of metabolism-related genes, and it even affects post-translational modifications of proteins such as acetylation and ubiquitination (Li et al., 2021). Furthermore, the role of lncRNA interactions with cell death in tumorigenesis, invasion, prognosis, and other aspects has been well-documented. An increasing number of studies have constructed predictive models of ferroptosis-related lncRNAs, which have shown great potential and significance in predicting tumor response and prognosis in various cancers (Xu et al., 2021a; Tang et al., 2022). However, the biological behavior and prognosis of cuproptosis-related lncRNAs in ccRCC has not been explored yet.

In this study, we will explore the relationship ccRCC, cuproptosis, and lncRNA. Firstly, based on the gene expression files of the ccRCC cohort. We studied 11 lncRNA that were associated with cuproptosis and had comparable prognostic significances for ccRCC patients. Using these 11 lncRNAs, we developed a risk prediction model that, upon ROC curve, C-index curve, and survival analysis, as well as other clinical indicators, shows great applicability in patients with ccRCC. Finally, we evaluated the role of these cuproptosis-associated lncRNAs in tumor immune escape and immunotherapy to predict possible target pathways that may hopefully play a role in the development of new treatment drugs and protocols for ccRCC.

## 2 Materials and methods

### 2.1 Data acquisition and processing

RNA sequence data and clinical information of renal clear cell carcinoma were obtained using the TCGA database (https://tcga-data.nci.nih.gov/), on 22 May 2022. The TCGA renal clear cell carcinoma cohort included 541 tumor samples and 72 non-tumor samples. Patients with incomplete records of follow-up information were not included in the sample analysis.

### 2.2 Identification of cuproptosis-associated lncrnas in renal clear cell carcinoma

First, 19 coding genes (mRNAs) associated with cuproptosis were obtained from the previous primary study. The expression levels of 19 cuproptosis-associated genes were obtained from the expression matrix of TCGA using “limma” R software packages. Next, to identify specific lncRNAs associated with cuproptosis, we used correlation tests (cor Filter = 0.1; *p*-value Filter = 0.05) to screen lncRNAs associated with cuproptosis in ccRCC. Subsequently, we performed correlation tests for the above coding genes and lncRNAs associated with cuproptosis. Sankey diagram and correlation heatmap were drawn with the help of “ggplot2″, “ggalluvial”, “tidyverse”, and “ggExtra” R software packages.

### 2.3 Model construction of cuproptosis -associated lncRNAs

Following the acquisition of differential cuproptosis-associated lncRNAs, we performed univariate Cox regression, LASSO, and multivariate Cox regression to assess the corresponding prognostic value. We selected the appropriate lncRNAs using LASSO analysis. Subsequently, we constructed a prognostic model for ccRCC using multivariate Cox regression analysis of these lncRNAs, associated with survival. Patients were divided into high and low-risk groups based on risk scores.

Risk score = $\sum_{i-1}^{n}\exp$ ∗βi. (β denotes coefficient value and exp denotes lncRNA level).

Kaplan-Meier and ROC curves were plotted with the help of the “Survival” and “SurvivalROC” R software packages, while the “scatterplot3d” R package plotted PCA R software packages.

### 2.4 Construction and verification of nomogram

Using the “ survival”, “regplot”, and “rms” software packages, nomograms were constructed from prognostic features that included clinical characteristics like age, sex, staging, and risk score, allowing the analysis of survival probabilities at 1, 3, and 5 years. The total score ranges from 150 to 400.

### 2.5 Gene mutation landscape

We obtained data from the TCGA dataset, including RNAseq data, mutated maf data, and clinical information of patients about ccRCC. They were downloaded and visualized using the maftools package.

### 2.6 Analysis of immune-related function

The ssGSEA analysis was performed first and the scoring was corrected. Heatmap visualization was performed using the “pheatmap” and “reshape2″ packages after difference analysis.

### 2.7 Immune escape and immunotherapy

Using the tumor immune dysfunction and exclusion (TIDE) algorithm (http://tide.dfci.harvard.edu/), based on TIDE score, the reaction to anti-PD-1 and anti-CTLA4 immunotherapy in the TCGA cohort can be forecast. If TIDE point is > 0, the sample is non-responsive to immune checkpoint inhibitors; if it is < 0, the sample is responding to immune checkpoint inhibitors. Finally, visualization analysis was performed with ggpubr package.

### 2.8 Screen for potential drugs for disease

According to the Cancer Pharmacosensitivity Genomics (GDSC) (https://www.cancerrxgene.org/), a list of drugs included in, a regression model was constructed using the PRRophetic algorithm. Using the R package “PRRophetic”, TCGA-ccRCC gene expression profiles and drugs in high- and low-risk subgroups were utilized to forecast half-maximal inhibitory concentrations (IC50). The smaller the IC50 of a drug, the stronger the drug’s efficacy at suppressing cancer cells.

### 2.9 Gene enrichment

To investigate the basic functions of potential targets, we analyzed the data using functional enrichment. Gene ontology (GO) is a broadly available tool for annotation of gene features, particularly molecular functions, biological pathways and cellular components. The Kyoto Encyclopedia of Genes and Genomes (KEGG) enrichment is a useful access source for investigating the information on genomic functions. To gain a better insight into the oncogenic role of mRNAs, the ClusterProfiler package was used to analyze GO functions and KEGG pathways. The bubble map was plotted using the ggplot2 package of R software; the heatmap map was plotted using the “pheatmap” package of R software.

### 2.10 RT-PCR

Total RNA extraction was performed as follows: RNA extraction was performed using the Trizol kit. Reverse transcription synthesis of cDNA was performed using RevertAid First Strand cDNA Synthesis Kitt (Thermo Fisher, United States). SYBR Premix Ex Taq (Takara, Japan) instrument was used for relative quantification of data. Relative expression was calculated using the 2^−ΔΔ^ method (GAPDH was used as an internal reference). primer sequences for SGMS1-AS1. Forward (5′-GGA​TGG​CGA​TGG​TCA​GGA​AA-3′), reverse (5′-TTG​GAG​AGA​GAG​TTG​CTT​GGT​G-3′); primer sequence for GNG12-AS1. Forward (5′-ACC​TGC​GGA​TAC​AGG​ACT-3′), reverse (5′-CCA​GAA​GCT​GAT​GGC​CGT​AT-3′); primer sequence for SMARCA5-AS1. forward (5′-CASGATGTTCCGTCTGCGTC-3′), reverse (5′-GAT​TCC​CGC​CGT​GAG​GTA​AG-3′).

## 3 Results

### 3.1 Identification of cuproptosis-related lncRNAs

**FIGURE 1:**
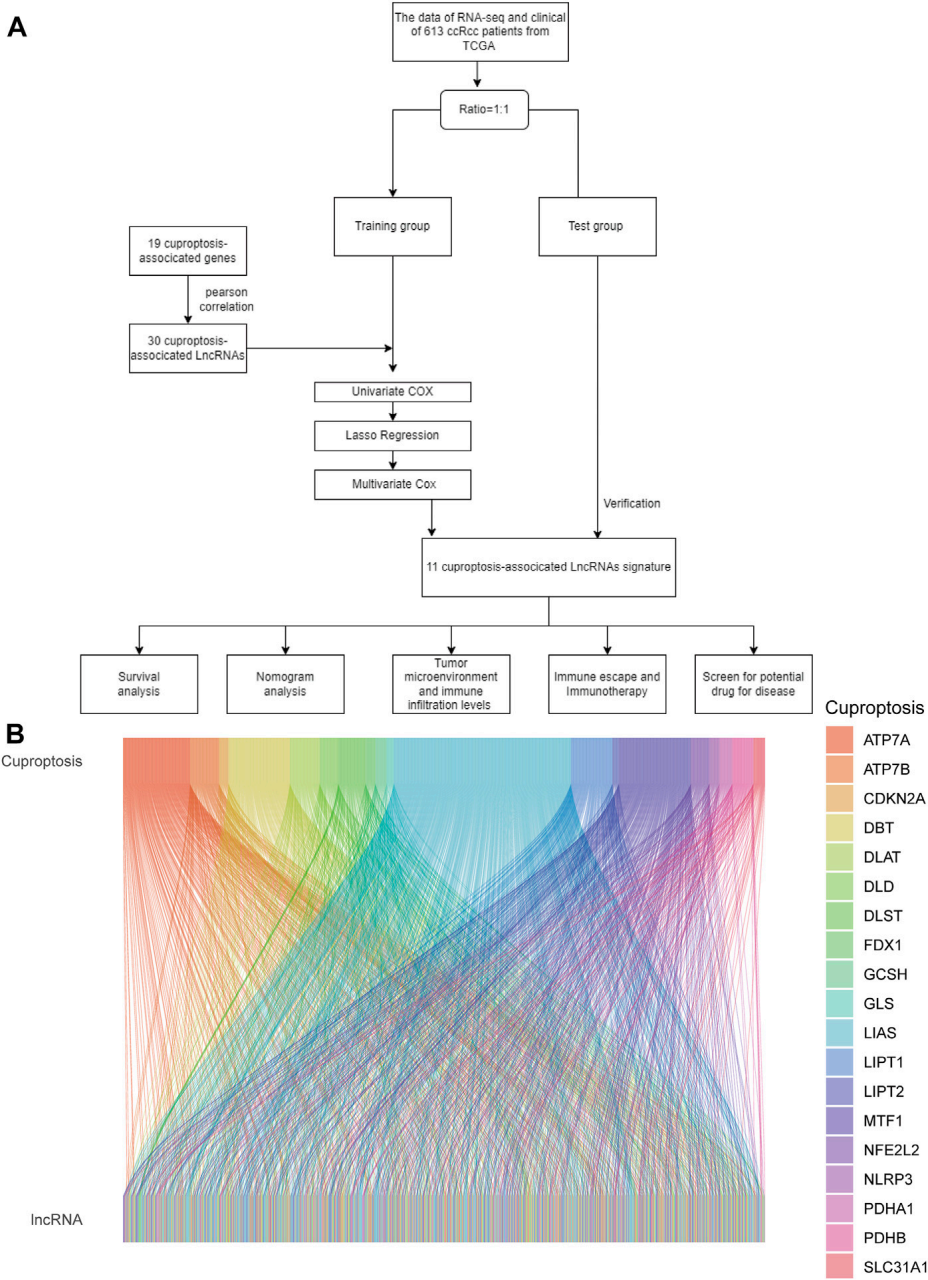
**(A)** Flowchart of the study. **(B)** Sankey diagram shows the association of lncRNAs associated with cuproptosis.

As shown in the flowchart in Figure 1A, we obtained 16,877 lcnRNAs from the ccRCC cohort using the TCGA database. 19 additional cuproptosis-associated genes were collected from available research. The relationship between ccRCC-associated lcnRNAs and cuproptosis-associated genes was visualized by the Sankey diagram and Pearson correlation analysis (Figure 1B).

### 3.2 Construction of risk score models

**FIGURE 2:**
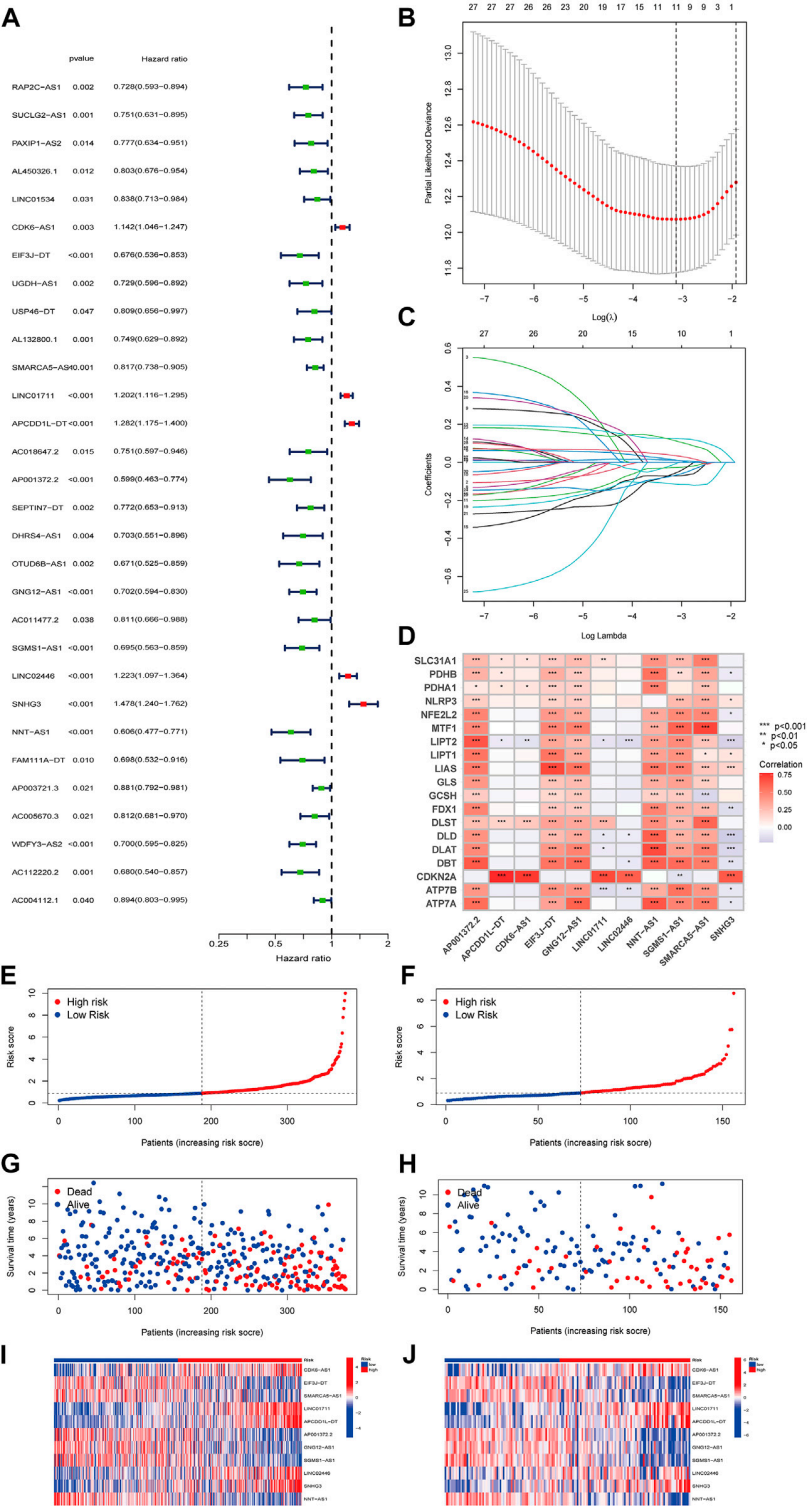
**(A)** Risk Forest Plot (red means high-risk lncRNA; green means low-risk lncRNA). **(B)** LASSO analysis of cuproptosis-related lncRNAs. **(C)** Cross-validation for tuning parameter selection in LASSO regression. **(D)** the correlation between cuproptosis genes and the 11 prognostic cuproptosis-associated lncRNAs in the proposed signature. The abscissa in the figure is lncRNA, ordinate is cuproptosis gene. **(E,F)** Distribution of risk score status in patients with train ccRCC and test ccRCC. **(G,H)** Scatterplot of survival status of patients with train ccRCC and test ccRCC. **(I,J)** Heat map of cuproptosis-related lncRNA expression profiles in train ccRCC and test ccRCC.

Univariate regression analysis revealed that 30 of the 55 cuproptosis-associated lcnRNAs screened from the ccRCC cohort, such as RAP2C-AS1, SUCLG2-AS1, PAXIP1-AS2, and LlNC01534, were strongly associated with the prognosis of ccRCC (Figure 2A). We then performed LASSO Cox regression to highlight the prognosis of the 11 lncRNAs associated with cuproptosis based on the lowest AIC (Figures 2B, C). Here, we interpreted the expression levels of each lncRNA to calculate the risk score = CDK6-AS1 × (0.0550731091024949)+EIF3J-DT × (−0.0994364819118698); SMARCA5-AS1 × (−0.0398456854170419) + LINC01711 × (0.026179353625897) + APCDD1L-DT × (0.128634099684908) + AP001372.2 × (−0.0654088007155913) + GNG12-AS1 × (−0.080630180236459) + SGMS1-AS1 × (−0.159906874521064) + LINC02446 × (0.0682696388752037); SNHG3 × (0.178418983910243) + NNT-AS1 × (−0.142242723024275). Cuproptosis-associated genes and these 11 cuproptosis-associated lncRNAs are closely related. For example, AP001372.2 was positively associated with LIAS, LIPT1, LIPT2 *etc.* APCDD1L-DT was positively correlated with PDHA1, PDHB *etc.* and negatively correlated with LIPT2. (Figure 2D). With the 11 cuproptosis gene-associated lncRNAs, we constructed a prognostic risk model to classify patients with ccRCC into high- and low-risk groups using the median risk score and validated the model with a test group. Risk curves show the correlation between the risk scores and risk levels. Patients were ordered based on the risk points of cuproptosis-associated lncRNAs. The risk was higher with higher scores (Figure 2E is the training; Figure 2F is the test). Scatter plots showed a strong correlation between survival duration and risk scores in ccRCC according to the cuproptosis-associated lncRNA model (Figure 2G is the training; Figure 2H is the test). Our results suggest that the risk score can more accurately predict and reflect the prognosis of ccRCC patients. The heat map then shows the relationship between the levels of cuproptosis-related lncRNAs in the model and the risk score (Figure 2I is the training; Figure 2J is the test).

### 3.3 Evaluation of the prognostic risk score model

**FIGURE 3:**
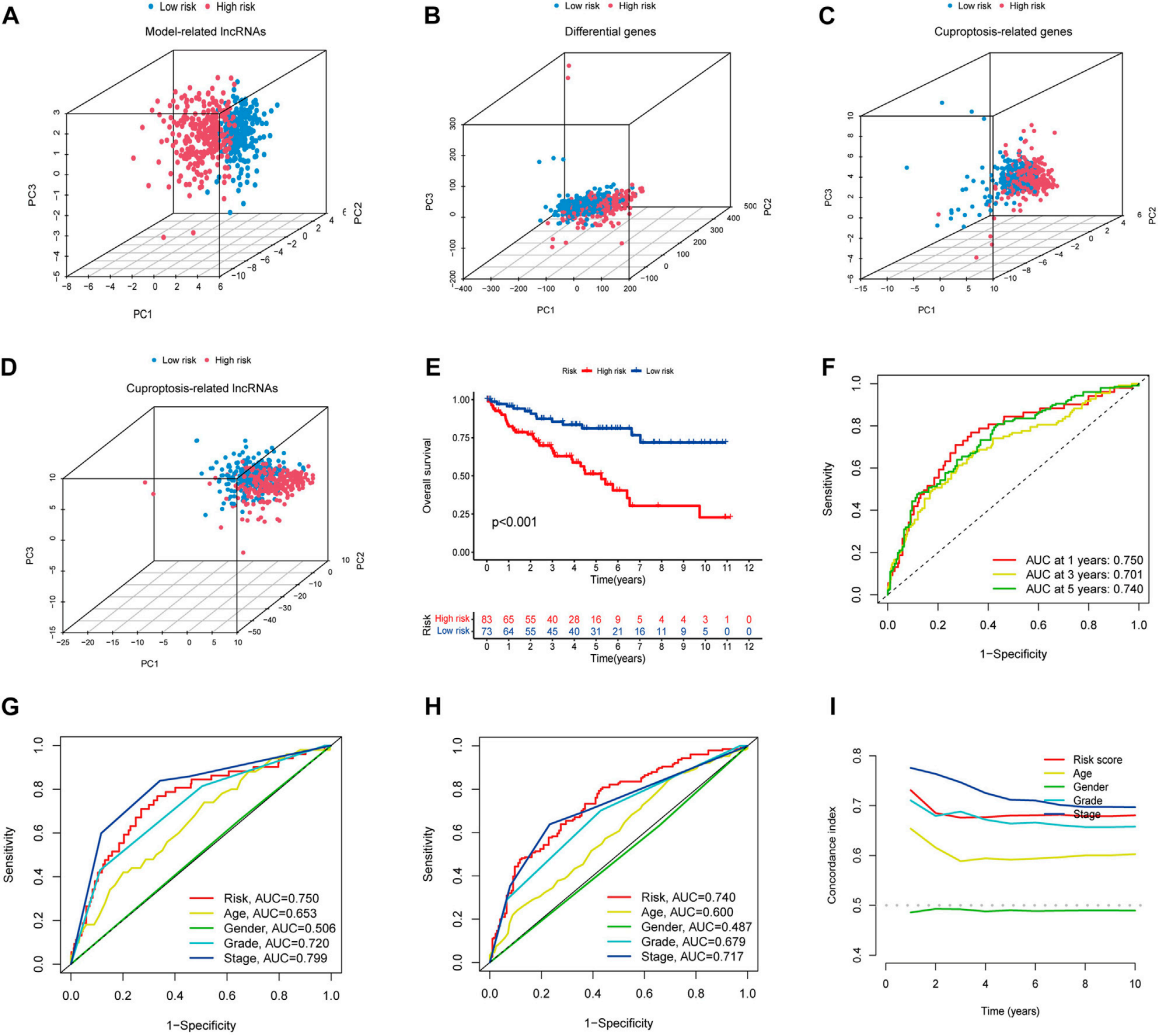
**(A–D)** PCA analysis of model lncRNAs, PCA analysis of cuproptosis-related lncRNAs, PCA analysis of cuproptosis-related genes, and PCA analysis of all genes, respectively. **(E)** Kaplan-Meier curves of high and low-risk group in TCGA. **(F)** ROC curves for 1, 3, and 5-year prognostic characteristics using CGGA risk score. **(G,H)** The ROC curves in TCGA data set. The larger the area under the curve, the greater the accuracy of predicting the survival time of patients through the model. **(I)** The C-index curve of risk score was used to assess the model. The ordinate is that the C-index score is the better the predictability.

PCA analysis showed that the lncRNAs involved in the construct model were able to most accurately distinguish between high- and low-risk groups compared to other indicators, such as various differentially expressed ccRCC genes, cuproptosis-related genes, and all cuproptosis-related lncRNAs (Figures 3A–D). Using Kaplan-Meier curves, we found that the survival rate of the high-risk group was significantly lower than that of the low-risk group (Figure 3E). The ROC curves showed that the constructed model had AUC values of 0.750, 0.701, and 0.740 at 1, 2, and 3 years, which indicated the remarkable predictive ability of the model (Figure 3F). The AUC value of the model was second only to tumor stage (AUC = 0.799) upon combined ROC was used to predict 1-year overall survival (OS) curve analysis and predict 5-year overall survival (OS) curve analysis, including age, sex, stage, grade, and risk score (Figures 3G, H). In addition, we further validated the constructed prognostic model by C-index curves and found the results to be as expected (Figure 3I). The results indicated that our model could evaluate the prognosis of patients with ccRCC.

### 3.4 Correlation of cuproptosis-related lncRNA models with clinical features

**FIGURE 4:**
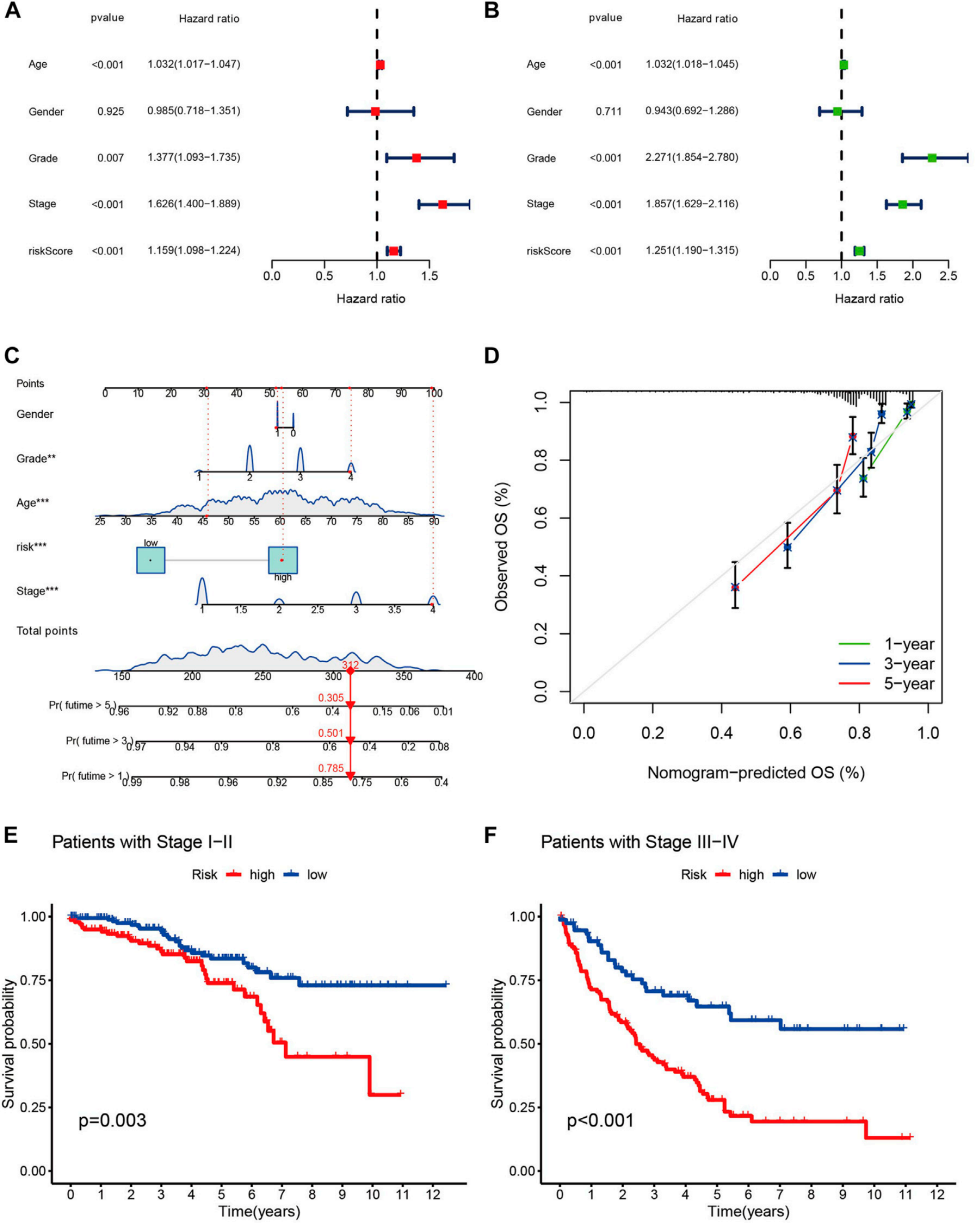
**(A,B)** The forest plots of *p*-value of univariate and multivariate Cox analysis of gene expression and clinical characteristics in TCGA, respectively. **(C)** Nomogram for combining clinicopathological factors and cuproptosis-associated lncRNAs for prediction. **(D)** Nomogram calibration curves. **(E,F)** the clinical survival of patients in the high- and low-risk groups of stage I-II and stage III-IV of ccRCC.

Univariate and multivariate Cox regression analysis clinical characteristics including age, sex, staging, and risk score showed that this model, constructed using 11 cuproptosis-associated lcnRNAs, was an independent factor for ccRCC clinical outcomes (*p* < 0.001) (Figures 4A, B). We designed a nomogram, including sex, age, staging, and risk score to predict 1, 3, and 5-year survival of patients with ccRCC. The calibration plots showed that this risk score performed well, that there was a good match between predicted and actual survival, and that the prediction model had a high predictive value (Figures 4C, D). Subsequently, we performed a stratified survival analysis to test the actual application of our prognostic risk model; the results showed that patients with stage I and II tumors with higher risk scores had a worse prognosis (*p* < 0.05). The same results were found in patients with stage III and IV tumors (*p* < 0.001) (Figures 4E, F). This suggests that the model is not only applicable to predict clinical outcomes in patients with early-stage, but also late-stage tumors.

### 3.5 Status and impact of tumor mutations in different risk groups

**FIGURE 5:**
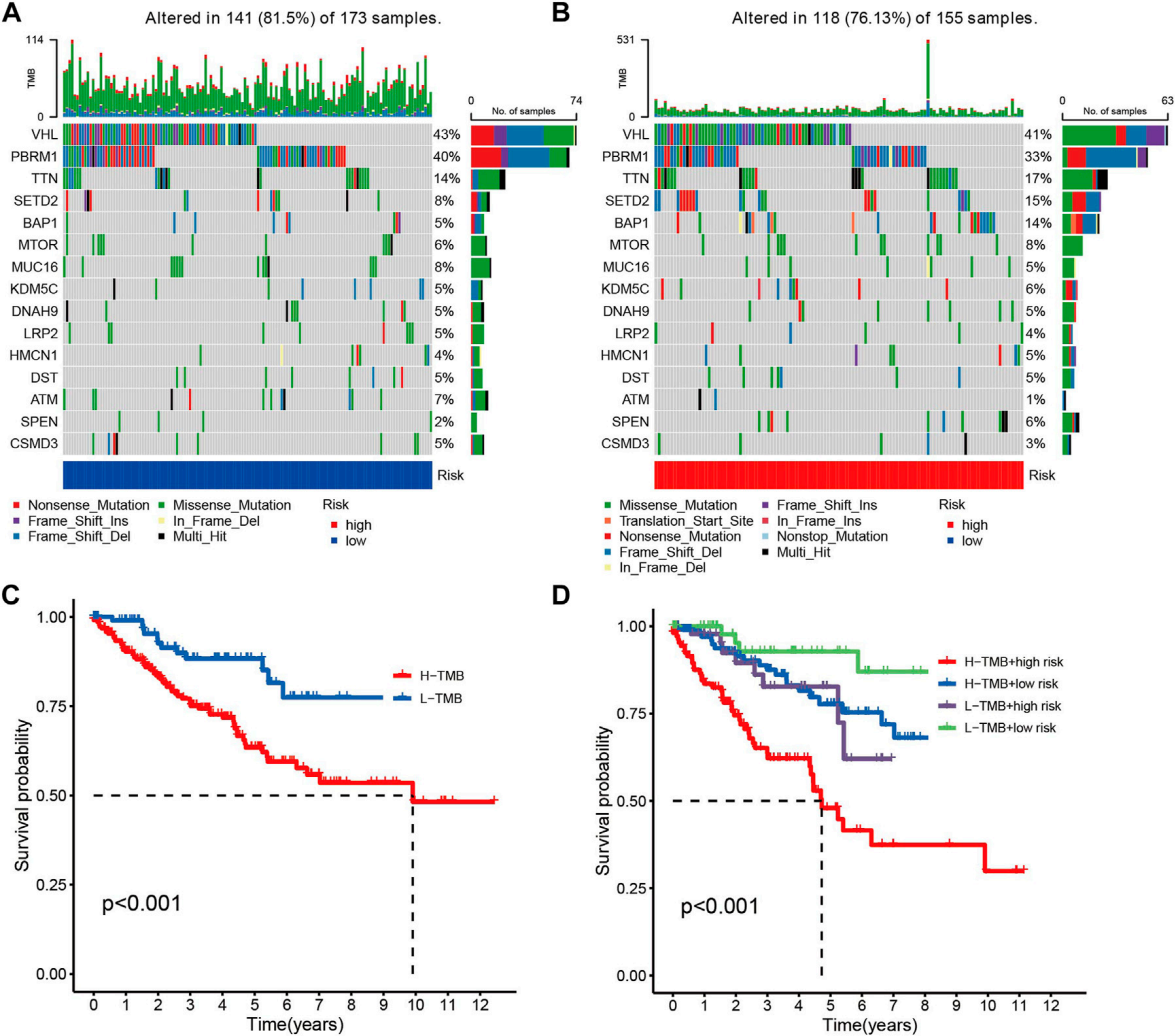
**(A,B)** Comparison chart of gene mutation frequencies. **(C)** Relationship between tumor mutation load and survival (red means high mutation rate, blue represents low mutation rate). **(D)** Relationship between TMB and risk score and prognosis.

Given the fact that the genetic mutations are an essential cause of oncogenesis, we investigated the distribution of somatic mutations. The 15 most common mutated genes in both groups are shown on the heat map. The overall mutation rate was similar in both high- and low-risk patients, but in the low-risk group, genes like VHL, PBRM1, and MUC16 showed a higher mutation rate in the low-risk group (Figures 5A, B). Kaplan-Meier curves for overall survival showed that patients in the high tumor mutation load (TMB) group had significantly lower OS than those in the low TMB group (Figure 5C). Combining tumor mutation load and risk score allowed us to classify patients into four groups. The combined survival analysis showed that the high tumor mutation load and high-risk groups had the worst prognoses, and, conversely, the low tumor mutation load and low-risk groups had the best prognoses (*p* < 0.001) (Figure 5D).

### 3.6 Immune escape immunotherapy tumor microenvironment and immune infiltration levelsBetween the two groups

**FIGURE 6:**
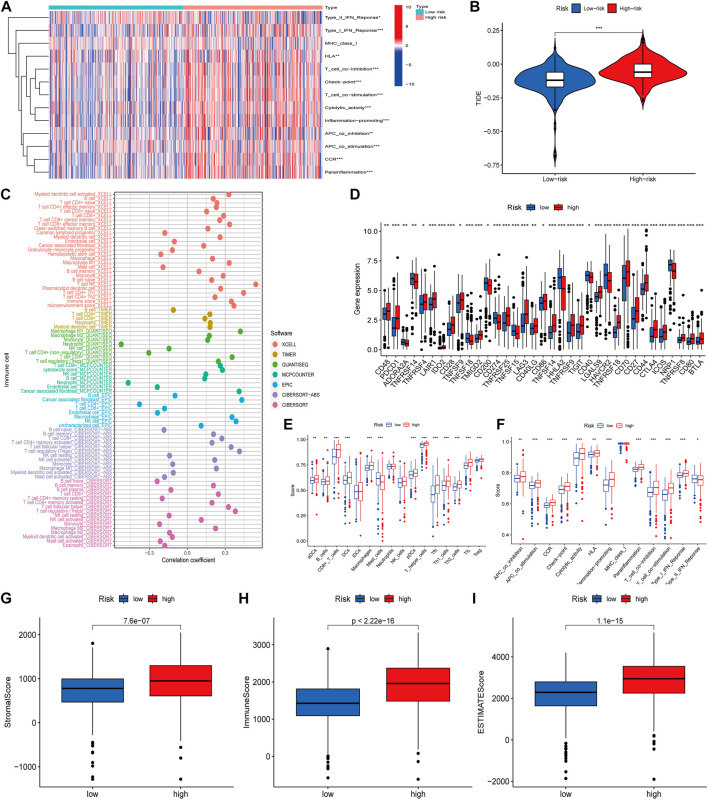
**(A)** Heat map of immune function differences. **(B)** Scoring of immune escape and immunotherapy in high- and low-risk groups. **(C)** Correlation coefficients between immune cells and risk scores, different colors representing different software predictions **(D)** Differential analysis of immune checkpoint related genes between high- and low-risk groups **(E,F)** Difference analysis of ssGSEA between immune cells and immune-related functions in high and low risk groups **(G–I)** The number of stromal cells, immune cell content, and comprehensive scoring of tumor microenvironment were different in the high- and low-risk groups (*p < 0.05; **p < 0.01; ***p < 0.001).

As immune checkpoint suppression therapy has become a focus of cancer treatment, we explored the role of risk models based on cuproptosis-associated lcnRNAs in predicting tumor-associated immunity As shown in Figure 6. Analysis of 13 immune-associated pathways showed that type I and II IFN response, HLA, checkpoint, co-stimulation, cytolytic activity, pro-inflammation, APC, CCR, and paraneoplastic inflammation were significantly different between high and low-risk groups (Figure 6A). Increased tumor immune dysfunction and rejection (TIDE) scores were detected more frequently in the high-risk group than in the low-risk group (Figure 6B); The results predicted by different software can visually show that immune cells such as B-cell naive and T-cell CD8 + are positively correlated with risk scores. Neutrophils are predominantly negatively correlated with risk score (Figure 6C). Immune checkpoint related genes analysis such as CD40 LGALS9 HAVCR2 TNFRSF18 CD70 were statistically significant in high- and low-risk groups (Figure 6D). Difference analysis of ssGSEA between immune cells and immune-related functions in high- and low-risk groups was mainly in CD8 + T-cell, T helper cells and other immune-related cells, Cytolytic HLA activity, and other immune functions (Figures 6E, F), and immune infiltration of the tumor microenvironment has been associated with improved survival for some patients with solid tumors. The number of stromal cells, immune cell contents, and the comprehensive scoring of the tumor microenvironment were significantly different between the high- and low-risk groups. These data support existing studies suggesting that in ccRCC tissue types, heterogeneous immune infiltrates are present, and low-risk groups show an improved prognosis (*p* < 0.001) (Figures 6G–I).

### 3.7 Screening for disease potential drugs and functional analysis

**FIGURE 7:**
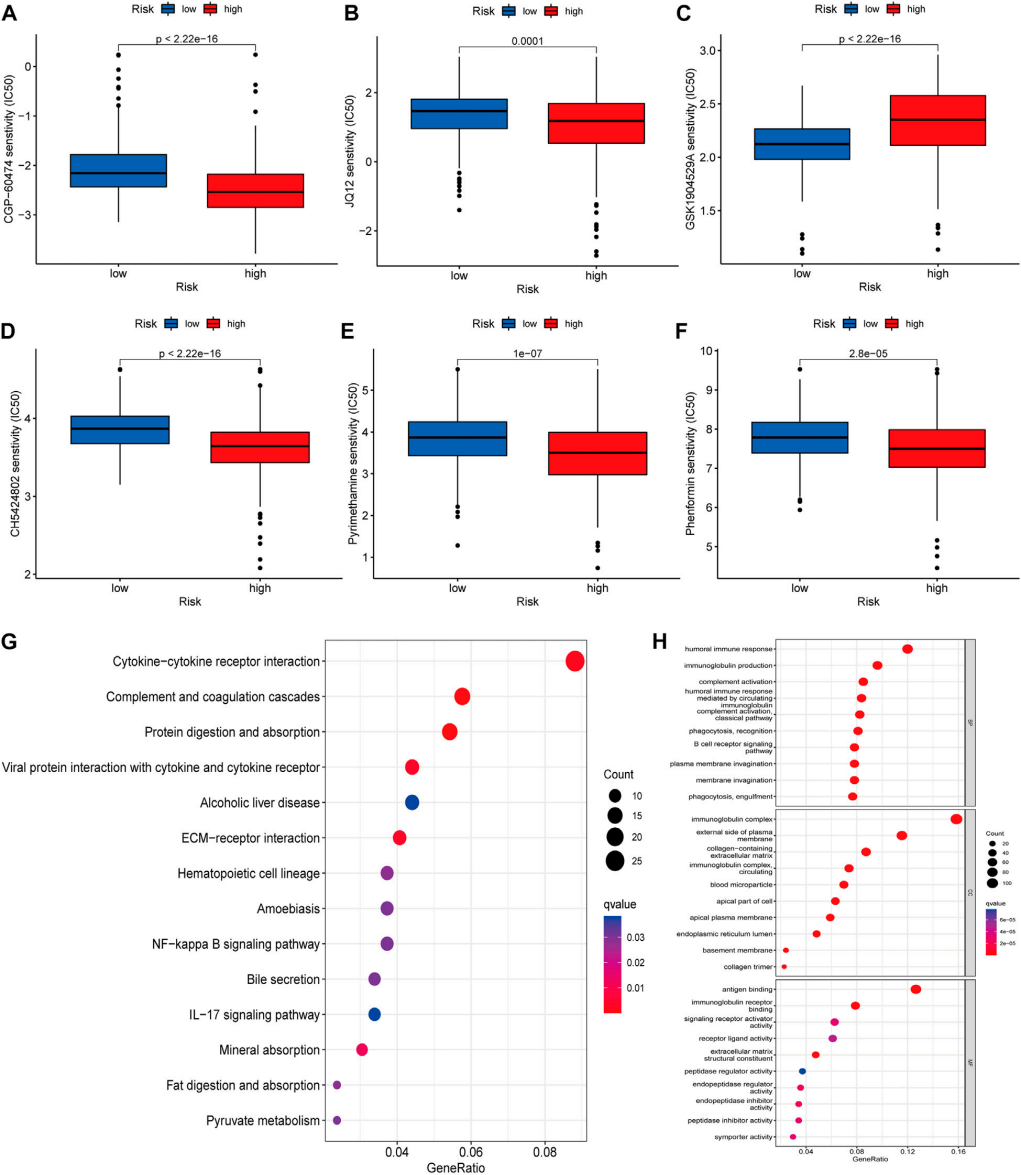
**(A–F)** The IC50 score in high- and low-risk drugs. Asterisks (*) stand for significance levels (*p < 0.05, **p < 0.01, ***p < 0.001) **(G,H)** KEGG and GO of differential genes in high- and low-risk groups, respectively Enrichment analysis, in which different colors represent the significance of differential enrichment results; the smaller the value of fdr, the more the number of circles. Circle size represents the number of enriched genes.

We using further drug sensitivity studies, we found that patients in the low-risk group were more sensitive to most immunosuppressive agents, such as CGP-6047A, JQ12, CH5424802, pyrimethamine, and phenformin, while the high-risk group only showed better sensitivity to GSK1904529A (Figures 7A–F). This means that patients from the lower risk group have a better chance of responding well to these chemotherapy drugs, and, subsequently have more treatment options. To further explore the mechanism of the role of cuproptosis-associated lcnRNAs in ccRCC, We performed GO and KEGG of differential genes in high- and low-risk groups. According to KEGG analysis, these RNAs were mainly involved in cytokine-cytokine receptor interactions, complement and coagulation cascades, protein digestion and absorption, alcoholic liver disease, EMC-receptor interactions, among others (Figure 7G). GO analysis was mainly involved in humoral immune responses, immunoglobulin production, immunoglobulin complexes, plasma membrane extrinsic, antigen binding, and immunoglobulin receptor binding (Figure 7H). These functions are vital to tumor the progression and immunotherapy response.

### 3.8 Validation of lncRNAs expression in ccRCC tissues

**FIGURE 8:**
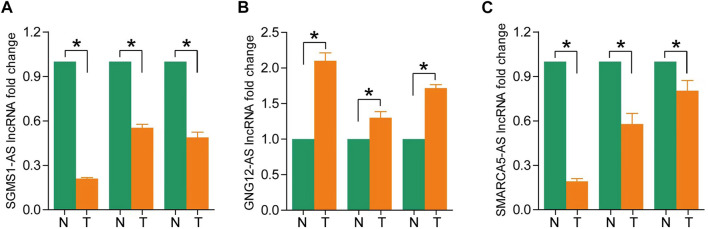
**(A–C)** Expression levels of SGMS1-AS1, GNG12-AS1, and SMARCA5-AS1 in ccRCC paraneoplastic tissues and ccRCC samples by RT-PCR (*p < 0.05, **p < 0.01, ***p < 0.001).

To assess differences in the expression of three landmark lncRNAs that constitute prognostic models in ccRCC and normal tissue, we used the unpaired Student’s *t*-test to examine expression levels of the three lncRNAs quantified by qRT-PCR. The qRT-PCR data of the three patients showed that lncRNAs SGMS1-AS1 and SMARCA5-AS1 were lower, and lncRNA GNG12-AS1 was higher in cancer tissues than in adjacent normal tissues. This further validated the accuracy of our previous bioinformatics analysis (Figures 8A–C).

## 4 Discussion

Renal cancer (RCC) is one of the most common tumors of the urinary system, and ccRCC is the most common (75%–80%) and best-studied subtype of RCC. Its treatment and management have always been challenging. Traditional radiotherapy and chemotherapy are essentially ineffective for RCC, and about 30% of RCC patients have metastatic disease at the initial visit, or relapse after complete resection of the primary tumor. Drug resistance of tumor cells in patients with relapse is the key to gauging the prognosis and determining future clinical action (Makhov et al., 2018). Although studies have shown that molecular targeting of vascular endothelial growth factor, platelet-derived growth factor, and inhibitor (PD1-PD-L1/CTLA4) to inhibit immune checkpoints can improve prognosis to a certain extent, tumor cells may grow immune to the new anti-tumor drugs. For example, the drug resistance of sunitinib and erlotinib is gradually increasing, and the overall survival rate of patients is still not optimistic (Barata and Rini, 2017). Therefore, the search for new immune checkpoints and targeted molecules is of great significance and requires urgency for the prognosis of ccRCC patients.

Cuproptosis is a recently defined unique mode of cell death. The mechanism of its occurrence mainly involves the homeostasis of copper ions (intake and output), mitochondrial respiration, energy metabolism—such as the TCA cycle, reduction of Fe-S cluster protein levels, the increase of HSP70 and other protein levels—as well as the triggering of protein toxicity. The researchers also identified key genes, such as FDX1, LIPT1, LIAS, DLD, NLRP3, GLS, among other key genes, associated with cuproptosis through genome-wide CRISPR-Cas9 loss-of-function methods. and ATP7B (Zheng et al., 2014; Tsvetkov et al., 2022). Although the impact of cuproptosis on tumorigenesis, development, prognosis, *etc.* remains to be further explored, the metabolic reprogramming involved in its genesis is based on its defining context in tumor cells and its changes in metabolic pathways of tumor energetics and biosynthesis (Xia et al., 2021). We can foresee that cuproptosis has a great predictive potential in the field of oncology. Related studies have demonstrated that ccRCC is also often accompanied by reprogramming of glucose and fatty acid metabolism, as well as TCA cycle dysfunction (Schaeffeler et al., 2019). Exploring the mechanisms associated with these metabolic pathways and exploiting the close link between cuproptosis and energy metabolism may contribute to the treatment of ccRCC.

The regulatory effects of lncRNAs on tumorigenesis, metastasis, and infiltration have gradually been comprehensively studied. The related mechanisms mainly include mediating post-translational modifications, regulating immune responses, promoting immune escape, and participating in metabolic reprogramming (Zhang et al., 2020; Li et al., 2021; Tan et al., 2021). Some studies have shown that lncRNAs regulate some elements of copper ion homeostasis and cuproptosis, such as SLC31A1 (CTR1), ATP7B, and GLS, and exert their influence on tumors. The lncRNA NEAT1 reduces the expression of CTR1 to regulate the function of cancer stem cells (Jiang et al., 2018). The lncRNA GLS-AS participates in the nutritional stress of pancreatic cancer by inhibiting GLS-mediated metabolism and controls tumor progression (Deng et al., 2019). In addition, lncRNAs themselves—or their encoded peptides—are also involved in the regulation of the carboxylic acid cycle of tumor cells (Stein et al., 2018; Sang et al., 2021). In summary, in tumor cells, lncRNAs regulate the expression of cuproptosis-related genes and the related mechanisms affect the occurrence and development of tumors. However, the role of cuproptosis-related lncRNAs on the treatment and prognosis of ccRCC tumors still needs further research.

In our study, we screened 55 lncRNAs associated with cuproptosis in ccRCC and constructed a model with the final selected 11 lncRNAs. These 11 lncRNAs have good discriminatory ability for high- and low-risk groups. There was a significant difference in OS between the high and low-risk groups. The 1, 3, and 5-year survival rates for this model corresponded to areas under the ROC curve that were all greater than 0.70. The results mentioned prior reflect the strength of the model in predicting the prognosis of ccRCC. The reliability of the model was further validated by the results of methods such as C-index, risk curve, calibration curve, and validation of clinical characteristics. Based on the above series of analysis, we found that most lncRNAs were protective in ccRCC patients, while only CDK6-AS1, LINC01711, APCDD1L-DT, LINC02446, and SNHG3 were independent risk factors for ccRCC. Studies have shown that LINC01711 is an independent prognostic factor for esophageal squamous cell carcinoma (ESCC) and, combined with other autophagy-related lncRNAs, can help forecast the prognosis and treatment of ESCC (Shi et al., 2021). The experiments carried out by the Mei-Ling Xu team proved that LINC01711 promotes the proliferation, migration, and invasion of ESCC (Xu et al., 2021b). The predictive effect of LINC02446 combined with other lncRNAs on tumor overall survival has been studied in bladder cancer and melanoma (Zhang et al., 2021a; Tong et al., 2021). The molecular mechanisms involved in bladder cancer mainly involve EIF3G-related translational activation as well as mTOR signaling pathway activation (Zhang et al., 2021b). SNHG3 plays a more prominent role in tumors, involving endogenous competing miRNAs, encoding peptides, *etc.* As an independent predictor of ccRCC, research relating to SNHG3 has recently improved (Yang et al., 2020). Topoisomerase II Alpha (TOP2A) is an enzyme that controls and changes the topological state of DNA, and can exert anti-tumor effects (Buzun et al., 2020). SNHG3 promotes ccRCC cell proliferation through a TOP2A-dependent pathway (Zhang et al., 2019). Baculoviral inhibitor of apoptosis repeat-containing 5 (BIRC5) is another regulatory target of lncRNA SNHG3 in ccRCC (Xu et al., 2021c). Cuproptosis-related lncRNAs can participate in the regulation of tumorigenesis and progression by binding to DNA, miRNA, and proteins, thereby affecting tumor progression, patient prognosis, and treatment outcomes. There is no study that examines the mechanisms of lncRNA and cuproptosis interactions in tumors. We established a cuproptosis-related lncRNA-based risk model to predict ccRCC and assist its early diagnosis and treatment.

The immune escape of tumor cells is a vital factor in tumor progression. Many studies have shown that lncRNAs can regulate tumor immune responses and promote tumor immune escape. Hepatocyte-derived exosomal lncRNA TUC339 is involved in cytokines, chemokines, Toll-like receptors and other related signaling molecules (Li et al., 2018). LncRNA MIAT is positively correlated with immune checkpoint molecules, such as PD-1 and CTLA4, in liver cancer cells (Peng et al., 2020). Therefore, we further carried out the immune-related analysis of 11 cuproptosis-related lncRNAs. We found significant differences in 13 immune-related pathways, TIDE scores, tumor microenvironment, and immune infiltration levels between high- and low-risk groups. These results suggest that lncRNAs associated with cuproptosis play a key role in regulating the associated immunity and patient prognoses in ccRCC cases. We analyzed the clinical sensitivity of immunotherapy drugs and found that the drug sensitivity of the high-risk group was significantly lower than that of the low-risk group, and that there were very few effective for the high-risk group; the low-risk group is more likely to have an effective immunotherapy course. In addition, in order to explore further functions of cuproptosis-related lncRNAs in ccRCC in the future, we performed GO and KEGG functional analysis, and found that most of these functions are related to tumor development and tumor immunotherapy. Finally, we detected lncRNAs in normal tissue and ccRCC tissue prognostic model using qRT-PCR method. These results confirmed the conclusions obtained earlier and made the study more accurate and complete.

There are several limitations in this study. First, we analyzed the prognostic role of cellular cuproptosis-related lncRNAs in ccRCC, but the interactions with target genes, signaling pathways, as well as themselves, needs further elucidation. In addition, our study verified the different expression levels of 3 representative lncRNAs associated with risk models quantified by RT-PCR. However, due to the difficulty of specimen collection, we did not measure the levels of all 11 cuproptosis-related lncRNAs.

In conclusion, our study marks the first analysis of cuproptosis-related lncRNA in ccRCC. We have exhaustively verified the predictive ability and reliability of the associated risk assessment model.

## Data Availability

Publicly available datasets were analyzed in this study. This data can be found here: TCGA.
